# Proteorhodopsin Overproduction Enhances the Long-Term Viability of Escherichia coli

**DOI:** 10.1128/AEM.02087-19

**Published:** 2019-12-13

**Authors:** Yizhi Song, Michaël L. Cartron, Philip J. Jackson, Paul A. Davison, Mark J. Dickman, Di Zhu, Wei E. Huang, C. Neil Hunter

**Affiliations:** a Department of Engineering Science, University of Oxford, Oxford, United Kingdom; b Department of Molecular Biology and Biotechnology, University of Sheffield, Sheffield, United Kingdom; c ChELSI Institute, Department of Chemical and Biological Engineering, University of Sheffield, Sheffield, United Kingdom; d Kroto Research Centre, University of Sheffield, Sheffield, United Kingdom; Shanghai Jiao Tong University

**Keywords:** proteorhodopsin, *Escherichia coli*, membrane, Raman spectroscopy, single cells, cell viability, marine bacteria, cell membranes

## Abstract

Genes encoding the photoreactive protein proteorhodopsin (PR) have been found in a wide range of marine bacterial species, reflecting the significant contribution that PR makes to energy flux and carbon cycling in ocean ecosystems. PR can also confer advantages to enhance the ability of marine bacteria to survive periods of starvation. Here, we investigate the effect of heterologously produced PR on the viability of Escherichia coli. Quantitative mass spectrometry shows that E. coli, exogenously supplied with the retinal cofactor, assembles as many as 187,000 holo-PR molecules per cell, accounting for approximately 47% of the membrane area; even cells with no retinal synthesize ∼148,000 apo-PR molecules per cell. We show that populations of E. coli cells containing PR exhibit significantly extended viability over many weeks, and we use single-cell Raman spectroscopy (SCRS) to detect holo-PR in 9-month-old cells. SCRS shows that such cells, even incubated in the dark and therefore with inactive PR, maintain cellular levels of DNA and RNA and avoid deterioration of the cytoplasmic membrane, a likely basis for extended viability. The substantial proportion of the E. coli membrane required to accommodate high levels of PR likely fosters extensive intermolecular contacts, suggested to physically stabilize the cell membrane and impart a long-term benefit manifested as extended viability in the dark. We propose that marine bacteria could benefit similarly from a high PR content, with a stabilized cell membrane extending survival when those bacteria experience periods of severe nutrient or light limitation in the oceans.

**IMPORTANCE** Proteorhodopsin (PR) is part of a diverse, abundant, and widespread superfamily of photoreactive proteins, the microbial rhodopsins. PR, a light-driven proton pump, enhances the ability of the marine bacterium *Vibrio* strain AND4 to survive and recover from periods of starvation, and heterologously produced PR extends the viability of nutrient-limited Shewanella oneidensis. We show that heterologously produced PR enhances the viability of E. coli cultures over long periods of several weeks and use single-cell Raman spectroscopy (SCRS) to detect PR in 9-month-old cells. We identify a densely packed and consequently stabilized cell membrane as the likely basis for extended viability. Similar considerations are suggested to apply to marine bacteria, for which high PR levels represent a significant investment in scarce metabolic resources. PR-stabilized cell membranes in marine bacteria are proposed to keep a population viable during extended periods of light or nutrient limitation, until conditions improve.

## Abstract (precis)

Proteorhodopsin (PR) is part of a diverse, abundant, and widespread superfamily of photoreactive proteins, the microbial rhodopsins. PR, a light-driven proton pump, enhances the ability of the marine bacterium *Vibrio* strain AND4 to survive and recover from periods of starvation, and heterologously produced PR extends the viability of nutrient-limited Shewanella oneidensis. We show that heterologously produced PR enhances the viability of E. coli cultures over long periods of several weeks and use single-cell Raman spectroscopy (SCRS) to detect PR in 9-month-old cells. We identify a densely packed and consequently stabilized cell membrane as the likely basis for extended viability. Similar considerations are suggested to apply to marine bacteria, for which high PR levels represent a significant investment in scarce metabolic resources. PR-stabilized cell membranes in marine bacteria are proposed to keep a population viable during extended periods of light or nutrient limitation, until conditions improve.

## INTRODUCTION

Microbial rhodopsins are ubiquitous in nature and comprise a diverse, abundant, and widespread protein superfamily of photoreactive proteins that either drive metabolic reactions or act as photosensors that carry out signaling and regulatory roles (1). Despite their varied roles, rhodopsins all comprise seven transmembrane α-helices that form a binding pocket for retinal (1). In 2000, Béjà et al. reported the existence of proteorhodopsin (PR), a rhodopsin found in several uncultured species of gammaproteobacteria (2). Subsequently, genes encoding PR were found in many different marine bacterial species (3, 4) and even viruses (5). PR is now believed to represent globally the most widespread phototrophic system at the genetic level (4). Thus, PR and chlorophyll-based photosystems represent two routes for harvested solar energy to power the biosphere (4).

The first report of PR established its function by expressing the PR-encoding gene, obtained from SAR86, in Escherichia coli. Exogenously supplied retinal was sequestered by the recombinant PR and formed a photoactive protein capable of initiating a photocycle and generating a transmembrane proton gradient (2). The means to produce functional PR, both natural and engineered variants, led to a proliferation of structural and biophysical studies, which established its oligomeric state, structural organization, and photocycle and established new fields of research that now encompass electrophysiology and optogenetics (reviewed in references 1 and 6).

Once it became possible to cultivate a marine PR-containing bacterium, the highly abundant SAR11 strain HTCC1062 (“*Pelagibacter ubique*”), it was shown that PR is a light-dependent proton pump, although it appeared to confer no increase in growth rate in the light (7). The effect of harboring PR on promoting cell growth has not been fully confirmed. PR-based phototrophy was demonstrated in the marine bacterium *Dokdonia* sp. strain MED134 (8), whereas the closely related *Dokdonia* sp. strain PRO95 had no growth advantage in the light, even though the PR gene was expressed at levels 10-fold higher in the light than in the dark (9). Deletion of the PR gene showed directly that PR phototrophy enhances the ability of the marine bacterium *Vibrio* strain AND4 to survive and recover from periods of starvation, lasting for up to 8 days (10). PR can also improve the survival of a host cell with no native PR, and it has been shown that PR extends the viability of Shewanella oneidensis strain MR-1 placed in nutrient-limited conditions over a 150-h period (11). Earlier work on E. coli had shown that heterologous production of PR, supplemented with exogenous retinal, allows illuminated cells to generate a proton motive force that powers the flagellar motor. Furthermore, cells containing PR and illuminated for 30 min had higher levels of survival in the presence of normally toxic levels of azide (12). Provision of a new energy source for the cell was one clear benefit of having PR; coexpression of the genes encoding PR and the retinal biosynthetic pathway yielded a strain of E. coli that could make the retinal cofactor and assemble a functional PR that created cells capable of photophosphorylation (13).

Here, we use Raman spectroscopy and imaging to examine the time-dependent assembly of PR in single cells of the heterologous host, E. coli; we quantify PR production using mass spectrometry and then show that populations of E. coli cells containing PR exhibit significantly extended viability over 41 days, with increased viability still measured after 9 months. Single-cell Raman spectroscopy (SCRS) detects the vibrational fingerprints of PR, nucleic acids, and membrane lipids in 9-month-old cells. This intriguing property of extended viability appears to be inherent to membrane assemblies of PR, which, as in marine bacteria, account for a large proportion of membrane area and represent a significant investment in metabolic resources. The results are consistent with marine bacteria using PR arrays in their membranes to extend the survival of the bacterial population during periods of severe nutrient limitation.

## RESULTS

### Detection of PR in single cells and real-time monitoring of PR assembly in E. coli.

**FIG 1:**
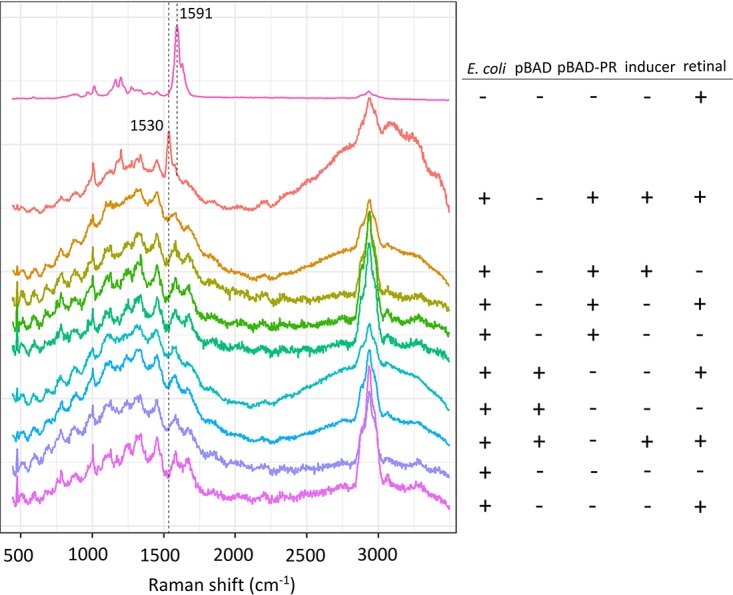
Single-cell Raman spectroscopy of PR in E. coli after induction of gene expression for 2 h. The top spectrum was recorded on pure retinal, in the absence of protein, and contained a characteristic Raman band at 1,591 cm^−1^. The Raman signal at 1,530 cm^−1^ (second spectrum from top) is indicative of retinal bound within PR. The remaining SCRS data were recorded on a series of negative controls, indicated by the plus and minus symbols on the right.

The plasmid-borne gene encoding PR was placed under the control of an arabinose-inducible promoter. After induction for 2 h in the presence of exogenously added retinal, E. coli cells expressing the PR gene became red, while the negative control with no plasmid remained a pale buff color. This observation is consistent with a previous report of PR production in E. coli (2). Here, we show that single-cell Raman spectroscopy (SCRS) is sufficiently sensitive to detect the expression of PR at the single-cell level. Figure 1 shows SCRS of E. coli cells induced for 2 h for expression of the plasmid-borne PR gene, as well as many other negative controls lacking either retinal, induction by arabinose, or a PR gene in the plasmid. SCRS of E. coli expressing the PR gene in the presence of retinal (Fig. 1, second spectrum from top) showed a band at 1,530 cm^−1^ that was not observed in any of the controls, including the spectrum for pure retinal. This signal, attributed to ethylenic stretching (*ν*_C=C_) vibrations in retinal-protein complexes, is consistent with Raman spectroscopy of purified PR protein (14); it provides a reliable biomarker for intact, functional PR and can be sensitively detected at the single-cell level, by using very weak laser power (0.15 mW) and 4 s of exposure. The weak laser excitation is essential to obtain a Raman signal of PR at the single-cell level, as higher laser power will quench PR.

**FIG 2:**
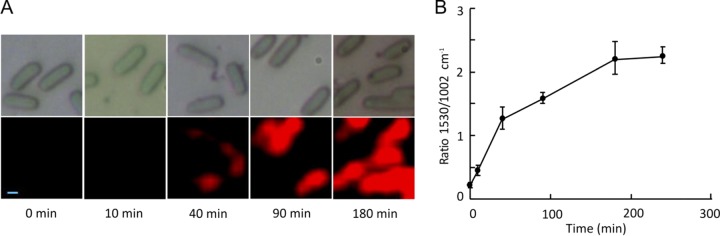
Dynamics of PR assembly in single cells of E. coli. (A) Series of optical (upper row) and Raman scanning (lower row) images recorded on cells at various times following induction of expression of the PR gene. The red color correlates with the intensity of the PR-specific band at 1,530 cm^−1^ in Fig. 1, normalized to the reference signal at 1,002 cm^−1^ for phenylalanine. Scale bar, 0.5 μm. (B) Graphical representation of the timescale for PR assembly. Error bars reflect the standard errors of results for between 41 and 167 single-cell replicates.

SCRS, specifically the PR band at 1,530 cm^−1^, was used to monitor the kinetics of PR assembly *in vivo* (Fig. 2). Since the cellular phenylalanine content is considered to be stable, the 1,002 cm^−1^ SCRS signal, assigned to phenylalanine, was used as a reference to compare the intensities of PR bands and to show PR assembly in single cells using a series of false color Raman images. Inspection of these Raman images in Fig. 2A (lower row) shows that PR assembly in the presence of exogenous retinal is visible after 40 min following induction of the plasmid-borne gene encoding PR. The 90-min image shows the widespread cellular distribution of PR, consistent with the high levels of this protein. Graphical presentation of the 1,530/1,002 cm^−1^ Raman signal ratio (Fig. 2B) shows that PR assembly is almost complete after 4 h of induction.

### Absolute quantification of PR production in E. coli.

**FIG 3:**
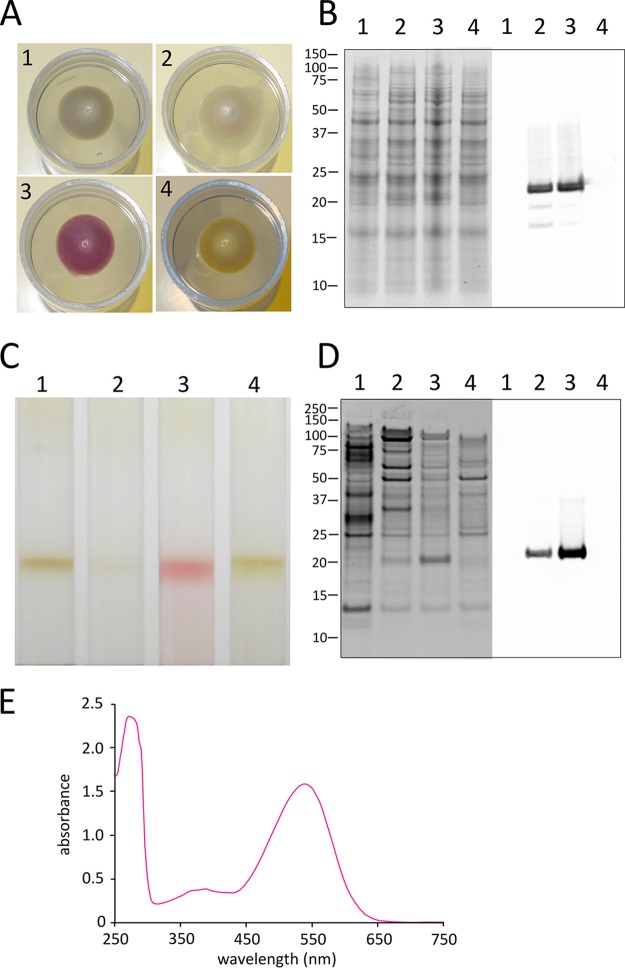
PR production by E. coli. (A to D) The samples are as follows: 1, E. coli BL21 PR, not induced; 2, E. coli BL21 PR, induced; 3, E. coli BL21 PR, induced, plus exogenously added retinal; 4, E. coli BL21 with no PR gene, induced, plus exogenously added retinal. (A) Images of cell pellets from 20-ml cultures. (B) SDS-PAGE (left) and immunoblot (right) analysis of whole cells, with detection of the C-terminal His tag on PR. Molecular masses of the markers are indicated on the left in kDa. (C) Purification of membranes on sucrose density gradients. Other details are as described for panel B. (D) Electrophoretic analysis of purified membranes, as described for panel B. (E) Absorbance spectrum of PR purified from membranes shown in panel C, gradient 3.

Growth of host E. coli cells was scaled up to a level where membranes could be purified to check levels of PR production. As expected, E. coli cells supplemented with retinal had a pink/purple color (Fig. 3A), reflecting abundant levels of His-tagged PR. The presence of the PR protein in these cells was verified by immunoblotting (Fig. 3B). Cells from all four cultures were lysed and then fractionated by rate zonal centrifugation; the presence of PR in the membrane fraction is shown by the colored band (Fig. 3C), the positive signal in the immunoblot of the membranes (Fig. 3D), and the absorption spectrum of the colored membrane. Both immunoblots (Fig. 3B and D) show that appreciable quantities of apo-PR are produced in cells induced in the absence of exogenous retinal. This finding is consistent with an earlier observation that PR is synthesized by E. coli in the absence of its cofactor (2).

Mass spectrometry in conjunction with stable isotope labeling was employed for absolute quantification of intracellular PR. Initial method development revealed that trypsin digestion of PR did not generate reliable proteotypic peptides (results not shown). We therefore used a formic acid digestion method which, when optimized, exhibits specificity for Asp-X and X-Asp sites (15), making this approach a viable chemical alternative to enzymatic proteolysis. ^15^N-labeled PR, prepared as described in the supplemental material, was added at a known concentration to E. coli cell lysates prior to formic acid digestion to generate peptide fragments for quantification by nanoflow liquid chromatography-tandem mass spectrometry (nanoLC-MS/MS). The acid digests were analyzed by nanoLC-MS/MS, and four peptide fragments mapping to both [^14^N]PR from the cells and [^15^N]PR from the internal standard were reproducibly identified by database searching, as shown in Fig. S1 in the supplemental material. Levels of PR, calculated as copy number per cell and shown in Table 1 (with additional details in Table S1), were derived as described in the supplemental material using the peptide ion counts shown in Tables S2 and S3. Negative-control analyses also included the same known concentration of a [^15^N]PR internal standard. E. coli, transformed with the pBAD plasmid lacking a PR insert, was devoid of isotopomer ion series mapping to [^14^N]PR peptides. As expected, only [^15^N]PR peptides from the internal standard were detected (Tables S2 and S3, columns A and B), indicating no production of PR. Similarly, analyses of E. coli containing the complete pBAD-PR plasmid but without arabinose induction showed only [^14^N]PR peptide isotopomer ions at background level (spectra not shown). Therefore, background PR expression was negligible (Tables S2 and S3, column C), confirming that PR expression is under a very tight control by the arabinose regulation system. Table 1 shows that, with arabinose induction, E. coli BL21 produces 186,868 ± 30,333 and 147,842 ± 20,113 PR copies/cell in the presence and absence of retinal, respectively (*P* = 0.001), broadly consistent with the immunoblots in Fig. 3B and D (tracks 2 and 3). Absorption spectroscopy revealed a holo-PR abundance of 177,000 copies/cell.

**TABLE 1:** Quantification of PR production in E. coli

Expt	Mean no. of PR copies/cell ± SD (*n* = 12)
pBAD, no PR gene, no arabinose, no retinal added	0
pBAD, no PR gene, arabinose, retinal added	0
pBAD + PR gene, no arabinose, no retinal added	0
pBAD + PR gene, arabinose and retinal added	186,868 ± 30,333
pBAD + PR gene, arabinose added but no retinal	147,842 ± 20,113

### Effects of PR overproduction on long-term viability of E. coli.

In view of the documented effects of PR on the viability of the marine bacteria *Vibrio* AND4 and Shewanella oneidensis strain MR-1, measured over durations of up to 8 days (10, 11), we designed experiments to test the viability and metabolic activity of E. coli cells containing the high levels of PR quantified in Table 1.

**FIG 4:**
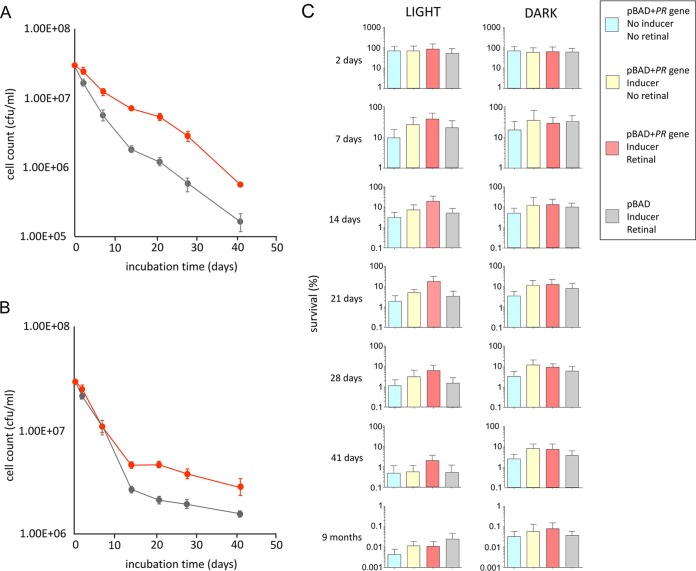
Effects of PR overproduction on long-term viability of E. coli. (A and B) Viability displayed graphically for the initial 41-day period. Results for E. coli with PR gene included on plasmid, retinal, and arabinose inducer present (red points) and E. coli with no PR gene but with retinal and inducer present (gray points) are shown for incubation in the light (A) and in the dark (B). (C) Histogram of cell survival over the full 9-month term of the experiment, in terms of absolute cell counts. Error bars are shown (*n* = 27). Blue, pBAD plus PR gene, no arabinose, no retinal; yellow, pBAD plus PR gene, arabinose, no retinal; red, pBAD plus PR gene, with arabinose and retinal; gray, pBAD only, with arabinose and retinal.

The viability of E. coli cultures containing PR was assessed for 41 days, with a final 9-month time point included. Controls excluded either the arabinose inducer, exogenous retinal, or the PR-encoding gene from the expression plasmid (see Materials and Methods), as follows: (i) E. coli (pBAD vector with the PR gene), no inducer, no added retinal; (ii) E. coli (pBAD vector with the PR gene) with arabinose inducer, no retinal; (iii) E. coli (pBAD vector with the PR gene) with arabinose inducer and supplemented with all-*trans*-retinal; and (iv) E. coli (pBAD vector only) with arabinose and supplemented with all-*trans*-retinal. Cells were kept at room temperature, either in the dark or under continuous illumination with low-intensity white light (5 μmol photons · m^−2^ · s^−1^) with three biological replicates for each condition, and each biological replicate was sampled in triplicate. Viability was assessed on the basis of counting CFU. The extents of survival over the initial 41-day period show the expected decline for all cultures, but for the first 21 days, E. coli (PR/retinal) in the light survives significantly better than control cells lacking PR (Fig. 4A). There was a smaller but still significant improvement for PR/retinal cells in the dark (Fig. 4B) than for the control.

The extents of cell survival from 41 days onwards are low and are therefore plotted in Fig. 4C on a logarithmic scale. In terms of absolute cell counts, the survival in the dark of 41-day cells containing PR, with (red bar in histogram) or without (yellow bar) added retinal, is significantly better than for cells with no PR (blue, gray). For the experiment in the light, the most viable 41-day sample was PR with the arabinose and retinal, which significantly outperformed the controls. Interestingly, all 41-day E. coli samples stored in the dark, including negative controls, were more viable than those in the light (Fig. 4A). This pattern continued, and after 9 months, cells maintained in the dark were more viable than those in the light. The combination of PR, arabinose, and retinal no longer conferred any benefit in the light after 9 months, but in the dark, PR-containing cells with (red) or without (yellow) retinal were more viable than those without PR (blue, gray).

### Longevity of holo-PR in E. coli cells.

**FIG 5:**
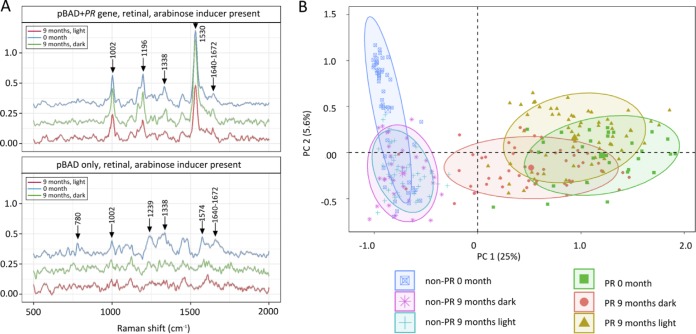
(A) Averaged Raman spectra of single cells of E. coli (pBAD plus PR gene, retinal, arabinose inducer present) and the negative control (pBAD only, with retinal and arabinose) following a 9-month incubation in the light (red) or the dark (green), with (upper) or without (lower) induction of expression of the plasmid-borne PR gene. (B) Score plot derived from a principal-component analysis (PCA) for a total of 302 single-cell Raman spectra, each comprising >1,500 Raman bands. Before input for the PCA, the spectra were normalized using the sum of amplitudes for the whole Raman spectrum for each cell.

SCRS allowed us to investigate the phenotypic profiles of individual cells from the extended viability experiment in Fig. 4, particularly in the presence of functional PR, as assessed by the 1,530 cm^−1^ Raman band. This is the same signal used to monitor the *in vivo* assembly of the PR-bound retinal chromophore in Fig. 2. Figure 5A shows SCRS of cells (plasmid-borne PR gene plus arabinose inducer and retinal) at the beginning and end of the 9-month incubation in either the dark or the light; the PR gene was absent from the negative control. Figure 5A (upper panel) shows that the 1,530 cm^−1^ band for the PR-bound retinal chromophore is still present after 9 months and absent as expected from the negative controls (lower panel).

As for Fig. 2, we used the 1,002 cm^−1^ SCRS signal, assigned to cellular phenylalanine content, to normalize the intensities of the PR signal at 1,530 cm^−1^. When 1,530/1,002 cm^−1^ ratios were calculated for a total of 142 single cells, there were no significant differences between the 0- and 9-month samples in the dark or the light (Fig. S2). The phosphate-buffered saline (PBS) medium used to suspend the cells contained no nutrients, so there was no cell growth and the PR detected after 9 months had originated from the protein produced at the outset of the experiment; thus, the 1,530/1,002 cm^−1^ ratio, a biomarker for PR, shows the longevity of PR *in vivo* over a time scale approaching 1 year.

We performed a more detailed and extensive analysis of the differences between the samples in Fig. 5 by measuring the amplitudes of more than 1,500 Raman bands recorded on 302 cells. This large data set was evaluated using principal-component analysis (PCA) and was used to dissect the effects of PR on 9-month-old cells after incubation in the light or the dark (Fig. 5B). The PCA in Fig. 5B shows that cells cluster in two distinct groups along the PC1 axis, one for PR cells at the start and end (0, 9 months) of the experiment and the other for non-PR cells. Within the latter category, Fig. 5B shows that SCRS of 9-month-old non-PR cells, both in the dark and the light, can be distinguished from the 0-month non-PR cells along the PC2 axis, indicating that the single-cell Raman spectra of the non-PR cells changed significantly over this long period.

**FIG 6:**
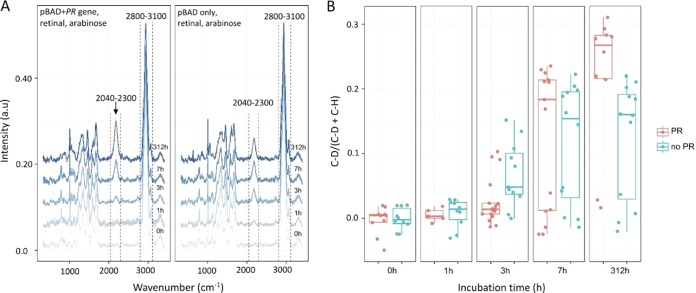
Effect of PR on deuterium incorporation by E. coli cells grown in the light. (A) Cells were incubated under nutrient-poor conditions (minimal medium with 35% D as D_2_O, supplemented with 1 mM glucose), and the SCRS of the cells was acquired at 0, 1, 3, 7, and 312 h of incubation. Each line represents the average of all recorded SCRS. (B) Box and whisker plot of the SCRS data compiled from 222 single cells. The C-D content was calculated from the 2,040-to-2,300 cm^−1^ area in the SCRS in panel A, and C-H was calculated from the 2,800-to-3,100 cm^−1^ area. The key is shown on the right: PR, cells with pBAD plus the PR gene, retinal, and arabinose inducer; no PR, the negative control with pBAD only, retinal, and arabinose.

The loading plot is shown in Fig. S3. Component 1 highlights the key differences between cells with and without PR, and the major band at 1,530 cm^−1^ in this plot arises from retinal bound within the PR protein. The clustering of points in Fig. 5B from 0-month and 9-month samples reflects the fairly constant PR content of E. coli cells over this extended period, as also seen in Fig. 5A. The component 2 plot (Fig. S3) indicates those factors involved in the 9-month cellular aging process, in the absence of PR. Of the many spectral features in this plot, the signals at 780, 1,239, 1,338, 1,574, and 1,640 to 1,672 cm^−1^ are usually assigned to stretching or vibrating modes from thymine, adenine, guanine, and cytosine rings, and the amplitudes of these signals were lower for the 9-month non-PR samples in the light and the dark than at the outset of the experiment, indicating reduced cellular levels of DNA/RNA. The 1,640-to-1,672 cm^−1^ region in SCRS is usually assigned to protein, and its decline in these non-PR cells is consistent with deterioration of cellular protein content during the long-term incubation with no nutrients present. Finally, the 1,420-to-1,470 cm^−1^ region in SCRS is usually assigned to unsaturated lipids, and its appearance in the PC2 loadings is consistent with deterioration of the cytoplasmic membrane in the cells lacking PR. In order to examine this point further, we compared the averaged amplitudes of Raman signals for 9-month-old cells with and without PR, in relation to cells at the outset of the experiment (Fig. S4). Signals in the 1,420-to-1,470 cm^−1^ region, assigned to lipids, were normalized to the 1,002 cm^−1^ signal for phenylalanine, taken here as a measure of protein content. There is a significant decrease in cellular lipid content in 9-month-old non-PR cells; it is likely that even a small reduction in lipid content could be deleterious for the cell, given the need to maintain a continuous, osmotically tight membrane bilayer. The lipids of bacterial membranes, for example, the fatty acid chain, alter in response to environmental changes (16–18). Although there is no direct relationship between lipid content and cell viability, it is reasonable to assume that maintaining a stable content of lipid should be beneficial. It has been reported that the fatty acid content of Vibrio vulnificus and E. coli membranes decreased greatly after long-term incubation at low temperature and viability, and the drop in membrane fatty acid content was correlated with the decreased bacterial viability (19). The production of unsaturated fatty acid in E. coli has also been proposed to enhance the robustness of the cells (20). In our study, we found that for the non-PR cell, the lipid content significantly dropped after the 9-month incubation (Fig. 5). Analysis of general metabolic activity shows that the non-PR cells (Fig. 6) were significantly lower in this respect than the cells with PR, implying that non-PR cells lose their stability.

Thus, we have identified at least some of the factors that contribute to the decline of 9-month-old cells lacking PR, and unsurprisingly they include essential structural components such as nucleic acids, protein, and lipids. We have also shown the long-term robustness of PR and a small but significant improvement in cell viability; this enhanced level of survival can be attributed to PR lowering the rate of deterioration in cell integrity.

### Increased metabolic activity of E. coli cells containing PR grown in the light.

A separate experiment was carried out at the single-cell level to investigate the possible benefits of PR conferred to cells grown in the light. Two cultures of E. coli were grown: one with pBAD plus the PR gene, retinal, and arabinose inducer and the negative control with pBAD only, retinal, and arabinose. The cells from each culture were washed and resuspended in minimal growth medium containing D_2_O and incubated (pBAD with or without the PR gene) at 37°C in the light. SCRS is able to quantitatively indicate general metabolic activity when cells are incubated with D_2_O (21). NADPD (deuterium D replacing H in NADPH) is generated in active cells because of an exchange of H with D in D_2_O. NADPD facilitates the formation of carbon-deuterium (C-D) bonds that display a distinguishable Raman band in an otherwise silent region (2,040 to 2,300 cm^−1^) in SCRS (Fig. 6A). To determine whether PR increases metabolic activity, SCRS of 10 to 20 single cells for each time point was recorded over a 312-h period. Incorporation of deuterium due to bacterial metabolism shifts the C-H bond signal from around 2,800 to 3,100 cm^−1^ to a C-D signal at 2,170 cm^−1^, visible in Fig. 6A (arrow) after 3 h. The extent of deuterium labeling, expressed as C-D/(C-D+C-H), was calculated for each SCRS for a total of 222 single cells and analyzed using a box and whisker plot (Fig. 6B). After 312 h, the extent of deuterium labeling in the PR-containing cells was significantly higher than that in the non-PR strain control (Fig. 6B). Thus, upon prolonged incubation under nutrient-poor conditions, cells can benefit from the extra source of energy provided by PR, which boosts the metabolism of the host cell.

## DISCUSSION

Our aim was to study the effects of PR in a bacterium that does not natively synthesize this protein, to use single-cell spectroscopy to quantify cell viability over long periods of weeks, even months, and to distinguish between the effects of apo-PR and holo-PR. We used Raman spectroscopy to observe the assembly of PR in single E. coli cells supplied with exogenous retinal, which achieved maximum levels over a period of 3 to 4 h following induction of gene expression (Fig. 2). Quantitative mass spectrometry showed that E. coli can synthesize high cellular levels of PR; when exogenous retinal was supplied, there were ∼187,000 PR molecules/cell, and quantitation by absorption spectroscopy gave a holo-PR abundance of 177,000 molecules/cell, in good agreement with the estimate from mass spectrometry. We tentatively conclude that the majority of PR is likely to be in the retinal-bound (holo) form. The mass spectrometry analysis showed that E. coli synthesizes ∼148,000 PR molecules/cell even in the absence of added retinal. The metabolic burden of synthesizing such a major nonnative cellular component could be expected to diminish cell viability, but we found the opposite: PR confers a clear benefit on its host cell. Over the initial 41-day period of our experiment, cells grown in the light with a functional PR were clearly more viable than the controls, whereas PR conferred only a small benefit in the dark (Fig. 4A and B). We included a long-term 9-month time point to show that irradiation over such a long period is generally deleterious; however, the cells grown in the dark continued the trend seen after 41 days, and cells with PR, with or without retinal, were more viable than the controls even though the absolute viability counts were very low (Fig. 4C). The Raman fingerprint of holo-PR was identified in SCRS of 9-month-old E. coli cells, incubated in the light or the dark (Fig. 5A). The remarkable stability of holo-PR is a likely basis for extended viability; SCRS shows that non-PR cells have lower cellular levels of DNA and RNA, and they exhibit a decline in the Raman signal specific for unsaturated lipids, so there is a likely deterioration of the cytoplasmic membrane, which can be alleviated by PR. Finally, a deuterium labeling experiment using SCRS showed that PR enhanced the metabolism of illuminated cells over a 13-day period (Fig. 6). Single-cell analysis by Raman spectroscopy indicates the dynamics of PR synthesis at the single-cell level, and it also reveals the phenotypic variations in an isogenic population. Figure 6A shows the average spectra (obtained from ∼50 SCRS), as well as the standard error (denoted by the shadowed area beside each line), from each population, showing that there are only very small variations. Interestingly, after remaining under the nutrient-poor condition for more than 7 h, two subgroups with high and low metabolic activities formed within the isogenic population (Fig. 6B). This suggests that these bacteria can maintain their cellular integrity under a nutrient-poor growth condition, but the activities of individual cells differ. The finding that PR helps cell survival regardless of the presence of light is statistically verified (Fig. 5 and 6) despite the heterogeneity in the populations.

We suggest that for cells under long-term cultivation under low-nutrient conditions, the high cellular levels of PR could stabilize the cytoplasmic membrane and thereby extend the viability of a population of cells. A substantial amount of the E. coli membrane is required to accommodate high levels of heterologously produced PR; if the membrane area occupied by a PR monomer is ∼11 nm^2^ (calculated from atomic force microscopy [AFM] data in reference 38) and the area of cytoplasmic membrane in an E. coli cell of 2 μm by 1 μm by 1 μm is ∼4.7 μm^2^, then 2.2 μm^2^ of cytoplasmic membrane, 47% of the total area, is needed to house the 187,000 holo-PR molecules we measured using mass spectrometry. In the absence of retinal, apo-PR still occupies 37% of the cytoplasmic membrane. Tens of thousands of PR molecules are likely to form densely packed arrays, and the myriad extensive intermolecular contacts between PR could help to maintain the integrity of the cell membrane and enhance cell viability. The same principles could apply to the vast populations of marine bacteria that naturally make PR. Several laboratories have used spectroscopic methods to quantify PR levels, and they found between 10,000 and 50,000 PR molecules/cell, very high numbers given the small size of bacteria such as SAR86 (Table 2). It appears that marine bacteria allocate a high proportion of membrane area (assuming no invaginated membrane structures) for housing PR, with percentages comparable to those for E. coli heterologously producing PR.

**TABLE 2:** Proteorhodopsin and membrane area in bacterial cells

Bacterium	No. of PR copies/cell	Cell dimensions (μm)	Membrane surface area (μm^2^)	% cell surface occupied by PR	Reference(s)
E. coli BL21	187,000 (holo); 148,000 (apo)	2.0 by 1.0	4.7	47 (holo); 37 (apo)	This work
SAR86	24,000	0.12 to 0.2 by 0.37 to 0.89^*a*^	0.32	84	3, 34, 35
SAR11 strain HTCC1062 (“*Pelagibacter ubique*”)	10,000	0.12 to 0.2 by 0.37 to 0.89	0.32^*b*^	37^*b*^	7, 34
*Winogradskyella* sp. strain PG-2	52,200	0.5 by 1.2	1.89	32	22, 36
Shewanella oneidensis MR-1	40,000	0.6 by 3.4^*c*^	1.51	7	11, 37

a Assumed to be similar in size to SAR11, as mentioned in reference 34.

b Based on an average cell size of 0.16 μm by 0.63 μm.

c Taken from reference 37, which reported rod-shaped cells of Shewanella oneidensis MR-1 grown at 22°C that were, on average, 0.61 ± 0.11 μm in diameter and 3.38 ± 1.7 μm in length.

When the PR-synthesizing cells are incubated under nutrient-limited conditions in abundant light for a relatively short period, the increased survival rate can be partially attributed to the increase in energy acquisition. As evidence, our deuterium labeling experiment shows that PR increases the metabolic activity of E. coli cells incubated in the light within the first 13 days (Fig. 6). The viable counts for the first 21 days also show that E. coli cultures containing PR display a higher survival rate in the light than in the dark (Fig. 4C). Similarly, Beja et al. pointed out that in abundant light, PR-based phototrophy must contribute significantly to the energetic requirements of bacteria such as SAR86 (2). PR phototrophy also enables the marine bacterium *Vibrio* AND4 to recover from periods of starvation lasting for up to 8 days (10), and Shewanella oneidensis strain MR-1 heterologously producing PR under nutrient-limited conditions is viable for ∼7 days (11). Nonetheless, for longer periods, it appears that the increased survival rate can be attributed mainly to stabilization of cytoplasmic membrane rather than increased energy acquisition; we observed that after 28 days, it was the E. coli grown in the dark that benefitted from PR (Fig. 4C).

The survival rates of E. coli in our long-term viability experiment are low, at ∼0.01%, and the survival gains conferred by PR are only of the order of 2- to 6-fold. Nevertheless, the availability of nutrients can vary widely in the oceans (23–25), and PR could make a valuable contribution to the survival of a sparse bacterial population starved of nutrients over extended periods. By extrapolating from the viability and SCRS experiments conducted here on E. coli, where heterologously synthesized PR can occupy up to 47% of the cytoplasmic membrane, we suggest that marine bacteria investing scarce resources in assembling high cellular levels of PR gain a long-term advantage beyond survival in the light. Even in the dark, where there is no obvious benefit conferred by PR, E. coli cells can maintain membrane integrity, and possibly as a consequence, the cell can retain its DNA and RNA. If the same principles apply to marine bacteria, similarly high levels of PR will improve their ecological fitness, even for bacteria in deep water, where light levels are very low or absent. The extreme longevity of PR arrays could therefore aid membrane integrity and promote survival during extended periods of nutrient depletion lasting weeks or even months, until a new pulse of nutrient enables a few surviving cells to restore the population. This study also identified a Raman biomarker at 1,530 cm^−1^ for holo-PR at the single-cell level, which would be useful to investigate PR-expressing bacteria *in situ* from the ocean. In addition to studying other marine phototroph biomarkers such as carotenoids that had been proposed previously (26, 27), we will be able to study the function of PR in a more ecologically relevant context in the future.

## MATERIALS AND METHODS

### Plasmids, strains, and growth of E. coli for membrane preparation.

The E. coli strain used in this study was BL21. The plasmid pBAD-PR for overexpression of PR in E. coli was a kind gift from Judith Armitage, Department of Biochemistry, University of Oxford, United Kingdom. This plasmid contains a PR gene (clone BAC31A8) from an uncultured bacterium originally isolated from a sample of Monterey Bay seawater (2, 3); the gene carries a 6×His tag at the C terminus. Single colonies of E. coli were inoculated into 6 ml of LB medium containing 100 μg/ml ampicillin and grown overnight at 37°C in the dark with shaking at 230 rpm. A 750-μl volume of the culture was used to inoculate 50 ml of LB medium/ampicillin in a conical flask and allowed to grow for 2 h at 37°C with shaking. Overexpression of PR was induced with 0.2% l-arabinose (final concentration), and 5 μg/ml (final concentration) all-*trans* retinal was added where appropriate. Cells were allowed to grow for a further 4 h before being pelleted and stored at –20°C. For larger-scale cultures, the overnight starter was used to inoculate 400 ml of LB medium (plus appropriate antibiotic) in a 2.5-liter conical flask and allowed to grow for 2 h at 37°C with shaking.

### Membrane preparation.

Cells harvested from a 400-ml culture were resuspended in 10 ml membrane buffer A (20 mM MOPS [morpholinepropanesulfonic acid], pH 7, 0.1 mM EDTA); a few grains of DNase I and lysozyme were added to the suspension together with 200 μl of a 1 M stock solution of MgCl_2_, and then the suspension was left to incubate at room temperature for 1 h. The cells were then disrupted by two passes through a French pressure cell at 18,000 lb/in^2^. The lysate was centrifuged at 16,000 × *g* for 25 min, and the supernatant was layered onto a discontinuous 15%/40% (wt/wt) sucrose density gradient made in buffer A and centrifuged in a Beckman Ti45 rotor at 53,000 × *g* for 10 h. The membrane band, present just above the 15%/40% interface (Fig. 2C), was collected with a micropipette and then stored at 4°C overnight or frozen at –20°C until required.

### Confocal Raman microspectroscopy for detection of proteorhodopsin and spectral processing.

Prior to Raman acquisition, cells were washed with deionized water to remove any of the growth medium which may interfere with observation and Raman detection. Cells were then resuspended in deionized water, and 1 μl of each sample was mounted onto an aluminum-coated slide (Shanghai d-band Medical Co., Ltd.). The Raman spectra were acquired as previously described (28, 29) using a confocal Raman microscope (LabRAM HR Evolution; HORIBA Scientific, London, UK) equipped with an integrated microscope (BX41; Olympus). A 100× objective (MPIanN, numerical aperture [NA] = 0.9; Olympus) was used to observe and acquire Raman signals from single cells. The Raman scattering was excited with a 532-nm Nd:YAG laser (Ventus; Laser Quantum, Manchester, UK). Raman measurements with 300-line/mm grating resulted in a spectral resolution of ∼2.5 cm^−1^. The detector was a –70°C air-cooled charge-coupled-device detector (Andor, Belfast, UK). The laser spot was located in the center of each individual cell, with the laser power on a single cell at about 0.15 mW. Acquisition times for Raman spectra were 4 s for single-cell measurements. In the Raman imaging measurement, a scanning area of 8 by 8 μm containing 1 to 5 intact bacterial cells was selected, with a scanning step of 0.5 μm.

During the kinetic expression of PR, the cells were incubated in a 37°C shaker at 200 rpm. One-hundred-microliter volumes of cells were sampled at 0, 10, 40, 90, 180, and 240 min after PR-expressing induction. The baseline of each spectrum was corrected using Labspec6 software (HORIBA Scientific, London, UK). The ratio of the intensity of peaks at 1,530 cm^−1^ (PR band) to 1,002 cm^−1^ (phenylalanine band) was calculated to evaluate the intensity of PR. For Raman imaging, the images were generated through classical least squares (CLS) fitting by setting the spectrum at a 240-min expression as a reference. For the SCRS experiment investigating the metabolic activity of E. coli cells containing PR, cells were illuminated with a cool white 1.5-W bulb containing seven individual LED bulbs.

### Immunoblotting.

Cells were pelleted by centrifugation (5,000 × *g*, 4°C, 5 min) and resuspended at 25 times the original density in PBS. Ten microliters of this cell suspension was diluted with 10 μl DNase (1 mg/ml in water) and 30 μl water before incubation at ambient temperature for 1 h. Four microliters of the DNase-treated cell preparation was mixed with 76 μl 1× lithium dodecyl sulfate (LDS) sample buffer (NuPAGE, Life Technologies) and incubated at 37°C for 30 min and then 42°C for 5 min. Twenty microliters was subjected to PAGE (12% 10-well NuPAGE gel, bis-Tris/MES-SDS system; Life Technologies) by following the manufacturer’s protocol, and immunoblotting with a rabbit anti-6×His primary antibody (A190-114A; Bethyl Laboratories) was performed as described previously (30).

### Absolute quantification of PR in E. coli cells.

Aliquots of medium contained 1.7 × 10^9^ cells, as determined previously using a calibration curve of optical density (OD) and cell number counting of CFU (31). Cells were pelleted by centrifugation as described above and resuspended to 0.1 ml in 2% (wt/vol) SDS, 60 mM dl-dithiothreitol. After the addition of 0.1 ml of 0.1-mm zirconia/silica beads (BioSpec/Thistle Scientific), the cells were lysed by heating them to 95°C for 90 s and then vortexing them for 30 s. The heating/vortexing cycle was repeated 3 more times. The lysate was tested for complete solubilization by the absence of a pellet after centrifugation at 16,000 × *g* for 60 s. For each assay, 30 μl of lysate (equivalent to 5.1 × 10^8^ cells) was mixed with 200 pmol ^15^N-labeled internal standard PR (30.6 μM, determined by *A*_280_, in 20 mM MOPS, pH 7.0, 0.6 M NaCl, 1% β-octylglucoside [see the supplemental material]). Proteins were precipitated using a 2-D clean-up kit (GE Healthcare) according to the manufacturer’s instructions and redissolved in 12.5 μl formic acid (≥98% purity; Fluka). Once the pellet had completely solubilized, 15 μl acetonitrile and 72.5 μl water were added, and acidic proteolysis was carried out at 95°C for 4 h (32). The digest was dried by vacuum centrifugation, and free amino acids/short peptides were removed using a C_18_ spin column (Thermo Scientific) by following the manufacturer’s instructions. After further vacuum centrifugation, the digest was dissolved in 0.1% (vol/vol) trifluoroacetic acid in 3% (vol/vol) acetonitrile, and 500 ng was analyzed by nanoflow liquid chromatography (Ultimate 3000 RSLCnano; Thermo Scientific) coupled to a Q Exactive HF mass spectrometer (Thermo Scientific) operating in data-dependent acquisition mode (nanoLC-MS/MS) with parameters as described in the supplemental material. Peptides originating from PR (^14^N from the cells and ^15^N from the internal standard) were identified by database searching. The amount of PR expressed in the cells was calculated from the relative intensities of the ^14^N peptide ions and their ^15^N-labeled counterparts.

### Quantification of holo-PR in E. coli cells by absorption spectroscopy.

PR-positive cells (2.4 × 10^9^, as determined previously [31]) that were induced/supplied with retinal (A) and not induced/not supplied with retinal (B) were suspended in 0.5 ml 20 mM Tris-HCl, pH 8, 200 mM NaCl containing 2% (wt/vol) *n*-dodecyl-β-d-maltoside. The suspensions were sonicated using 5-s-on/5-s-off cycles at 30% power for 60 s, repeated a total of three times with cooling on ice for 5 min between each 60-s cycle. The lysates were then clarified by centrifugation at 5,000 × *g* for 10 min. Absorption profiles were acquired from 300 to 1,000 nm, and a difference profile was generated by subtracting B from A for holo-PR quantification at 520 nm using the extinction coefficient 50,000 M^−1 ^cm^−1^ (3).

### Viability of E. coli expressing the gene encoding PR.

Cells were grown, induced, and overexpressed in LB medium as described earlier. Cultures representing each of the four conditions were prepared for a long-term viability test: (i) uninduced E. coli (pBAD vector with the PR gene), no inducer, no added retinal; (ii) E. coli (pBAD vector with the PR gene) with arabinose inducer but no added retinal; (iii) E. coli (pBAD vector with the PR gene) induced by arabinose and supplemented with all-*trans*-retinal, and (iv) E. coli (pBAD vector only), but with arabinose and supplemented with all-*trans*-retinal. After being induced for 4 h, cells from cultures 1 to 4 were washed in PBS and each pellet was resuspended in a final PBS volume of 20 ml, to an OD_600_ of ∼4 (1-cm pathlength) in six transparent universal tubes (30 ml, Sterilin; Thermo Fisher, UK), giving 24 tubes in total. Twelve tubes (conditions i to iv above, each in triplicate) were kept in the dark at room temperature, while the other 12 (again, conditions i to iv, each in triplicate) were illuminated with constant light (∼3 μmol · m^−2^ · s^−1^) from a 15-W Megaman energy-saving bulb (warm white, 3,000 K), also at room temperature. The tubes were inverted every other day to mix the cells and medium. Cell samples (each 100 μl, in triplicate from each tube) were taken over the 9-month viability trial under strict sterile conditions. Serial dilutions were then performed, and 100 μl of each dilution was spread on LB agar plates to obtain the CFU numbers. For each of the three biological replicates, three samples were taken for serial dilution and counts were taken for three consecutive serial dilutions, giving 27 counts for each time point.

### Metabolic activity analysis of PR-expressing E. coli with Raman spectroscopy.

To evaluate changes in the metabolic activity of E. coli cells containing PR, we applied the Raman deuterium isotope probing (Raman-DIP) technique (21, 33). Briefly, two cultures of E. coli were grown, one with the pBAD plasmid with the PR gene, retinal, and arabinose inducer, and the negative control with pBAD only, retinal, and arabinose. After 4 h of induction to yield high levels of PR, the cells from each culture were washed and resuspended in minimal growth medium containing D_2_O (35%) and 1 mM glucose and incubated at 37°C in the light. At 0, 1, 3, 7, and 312 h after D_2_O addition, 50-μl samples of culture were removed and washed with deionized water. Single-cell Raman spectra were acquired as detailed above, but with the laser power set to 5 mW.

## Supplementary Material

Supplemental file 1
