# Supplementary material for: Proteorhodopsin Overproduction Enhances the Long-Term Viability of Escherichia coli
Source: Appl Environ Microbiol. 2019 Dec 13;86(1):e02087-19. doi: 10.1128/AEM.02087-19 (PMC6912077; doi:10.1128/AEM.02087-19)
Supplement: Supplemental file 1 [file AEM.02087-19-s0001.pdf]

Overproduction of proteorhodopsin enhances long-term viability of *Escherichia coli*

Yizhi Song, Michaël L. Cartron, Philip J. Jackson, Paul A. Davison, Mark J. Dickman, Di Zhu, Wei E. Huang, C. Neil Hunter

SUPPLEMENTAL FILE (pdf)

Chemicals for absolute quantification of PR expression by mass spectrometry.
Preparation of ^{15}N-labelled PR internal standard.
Identification of PR peptides.
Absolute quantification of PR expression.
FIG S1 Sequence coverage of PR peptide fragments generated by formic acid co-digestion of *E. coli* cell lysate and [^{15}N]PR-His_6 internal standard.

FIG S2 Box and whisker plot showing the cellular PR content, as measured by 1530:1002 cm^{-1} ratios, calculated for a total of 142 single cells.

Chemicals for absolute quantification of PR expression by mass spectrometry. All salts were analytical reagent grade and other chemicals were ≥ 98% as supplied by Sigma-Aldrich. Solvents for nanoLC-MS/MS and upstream sample processing were HPLC grade as supplied by Fisher unless stated otherwise.

Preparation of ^{15}N-labelled PR internal standard. *E. coli* cells carrying the pBAD-PR plasmid were grown and induced with L-arabinose as described in Materials and Methods (main article) except that the medium was M9 containing $(^{15}NH_4)_2SO_4$ (Cambridge Isotope Laboratories, 99%). 5 µg/ml all-*trans* retinal was also added to enable monitoring of subsequent purification of [^{15}N]PR-His_6 at 520 nm. Following initial isolation using IMAC (Cartron *et al.*, 2014), [^{15}N]PR-His_6 was concentrated to 3 ml using a Centriprep YM-50 ultrafiltration device (Millipore) and purified by size-exclusion chromatography on a Superdex 200 16/600 column in 20 mM MOPS pH 7.0, 0.6 M NaCl, 1% β-octylglucoside at 0.4 ml/min. Fractions absorbing at both 280 and 520 nm were pooled, concentrated as above and stored at -20 °C. For quantification, a 50 µl sample was treated with a 2-D Clean-up Kit (GE Healthcare) according to the manufacturer's instructions and the resultant protein pellet dissolved in 8 M urea before quantification by Nanodrop analysis (Thermo Scientific)

using its calculated extinction coefficient at 280 nm (75860 $M^{-1}.cm^{-1}$, A_{280} (1g/L) = 2.495, www.expasy.org/protparam). The protein concentration of 30.6 µM determined at 280 nm was comparable to 39.5 µM determined at 520 nm using the published extinction coefficient of 50000 $M^{-1}.cm^{-1}$ for PR-bound retinal (Ref 7, main article). The lower value was used for quantification by mass spectrometry.

Identification of PR peptides. 500 ng of total *E. coli* cell formic acid digest from all experiments: (A) PR^- / L-arabinose$^-$ / retinal$^-$, (B) PR^- / L-arabinose$^+$ / retinal$^+$, (C) PR^+ / L-arabinose$^-$ / retinal$^-$, (D) PR^+ / L-arabinose$^+$ / retinal$^+$ and (E), PR^+ / L-arabinose$^+$ / retinal$^-$ containing co-digested [^{15}N]PR-His_6 internal standard, were analysed (three technical replicates) by nanoLC-MS/MS. The LC delivered a linear gradient of 97% solvent A (0.1% formic acid in water) to 10% solvent B (0.08% formic acid in 80% acetonitrile) over 5 min followed by 10% - 50% solvent B over 3 h at 300nL/min. The Q Exactive HF mass spectrometer (Thermo Scientific) was programmed for automated data dependent acquisition with each full scan at 120000 resolution (automatic gain control target: 1.0 x 10^6, maximum injection time: 60 ms) followed by a maximum of ten dependent product ion scans at 30000 resolution (automatic gain control target: 1.0 x 10^5, maximum injection time: 60 ms). Data-files were converted to Mascot Generic File (MGF) format using MSConvert (proteowizard.sourceforge.net/download.html) and the MGFs used as input for searching using Mascot Daemon v. 2.5.1 running with Mascot Server v. 2.5 (Matrix Science) against the *E. coli* BL21-DE3 reference proteome database (www.uniprot.org/proteomes/UP000002032, 4822 proteins, downloaded on 9 November 2017) with the PR-His_6 sequence (Fig. S1) added. Search parameters were: formic acid digestion (user-defined as cleaving at both N- and C-terminal sides of Asp); maximum missed cleavages: 3; MS and MS/MS tolerances: 0.05 Da; no variable modifications; quantification: ^{15}N metabolic.

Absolute quantification of PR expression. 16 theoretical proteotypic peptides (6 – 30 residues in length) can be generated from PR-His_6 by proteolysis with formic acid which cleaves on both N-terminal and C-terminal sides of aspartic acid residues. Owing to this cleavage pattern, the peptides overlap and represent five sequence regions as shown in Fig. S1. Database searching (see above) identified four peptides at $p \leq 0.01$ mapping to two sequence regions (S90 – D98 and G214 – D228) indicated in red in Fig. S1. As expected, these peptides were detected in both ^{14}N and ^{15}N forms (from the *E. coli* cells and isotopically-labelled internal standard respectively) in experiments D and E (see above) in which the pBAD-PR plasmid was present and expressed. Similarly, only the ^{15}N forms of the peptides were identified in the negative control experiments A – C, containing

only the internal standard. The ^{14}N and ^{15}N peptide isotopomer ion series were extracted from the mass spectrometer data-files using the Thermo Xcalibur Qual Browser application v. 3.0.63 to derive spectral peak ion counts (Tables S2 and S3). The ^{14}N:^{15}N ratios were used with the cell counts to calculate PR expression levels as copy number per cell (TableS1).

Reference:

Cartron ML, Olsen JD, Sener M, Jackson PJ, Brindley AA, Qian P, Dickman MJ, Leggett GJ, Schulten K, Hunter CN. 2014. Integration of energy and electron transfer processes in the photosynthetic membrane of Rhodobacter sphaeroides. Biochimica Et Biophysica Acta-Bioenergetics 1837:1769-1780.

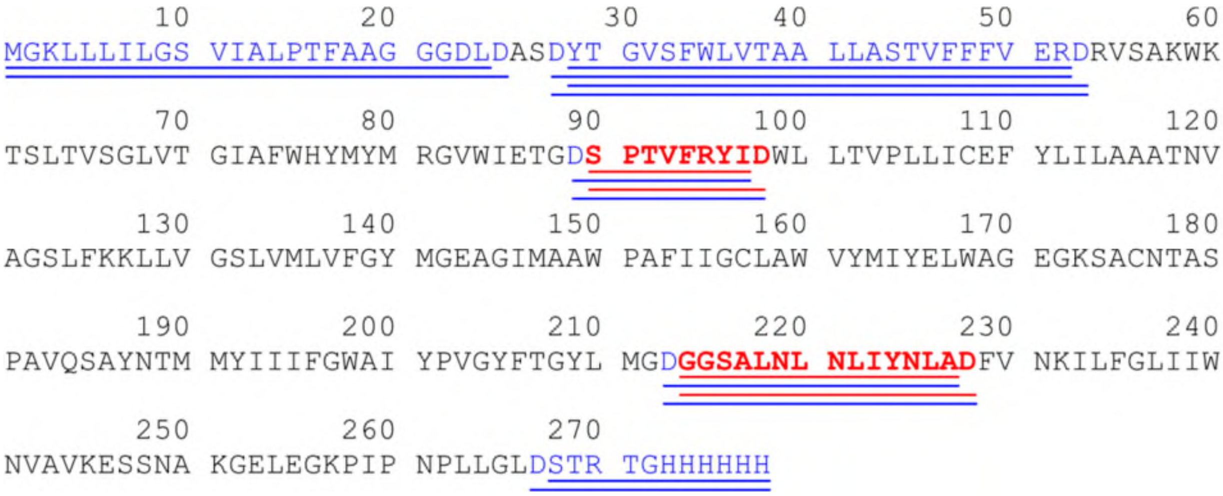

FIG S1 Sequence coverage of PR peptide fragments generated by formic acid co-digestion of *E. coli* cell lysate and [^{15}N]PR-His$_6$ internal standard.

The four peptides meeting the criteria for quantification were identified by the Mascot database search engine at $p \leq 0.01$ in both ^{14}N and ^{15}N forms and are indicated in red. Theoretical proteotypic peptides that were either not detected or were identified at $p > 0.01$ are shown in blue.

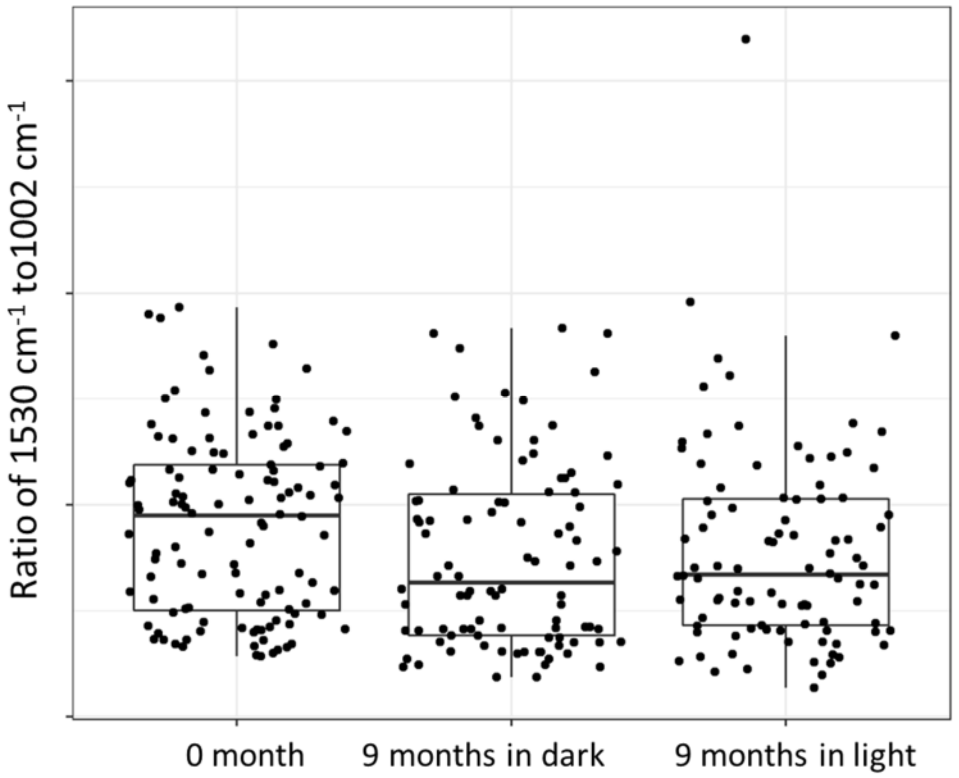

FIG S2 Box and whisker plot showing the cellular PR content, as measured by 1530:1002 cm^{-1} ratios, calculated for a total of 142 single cells.

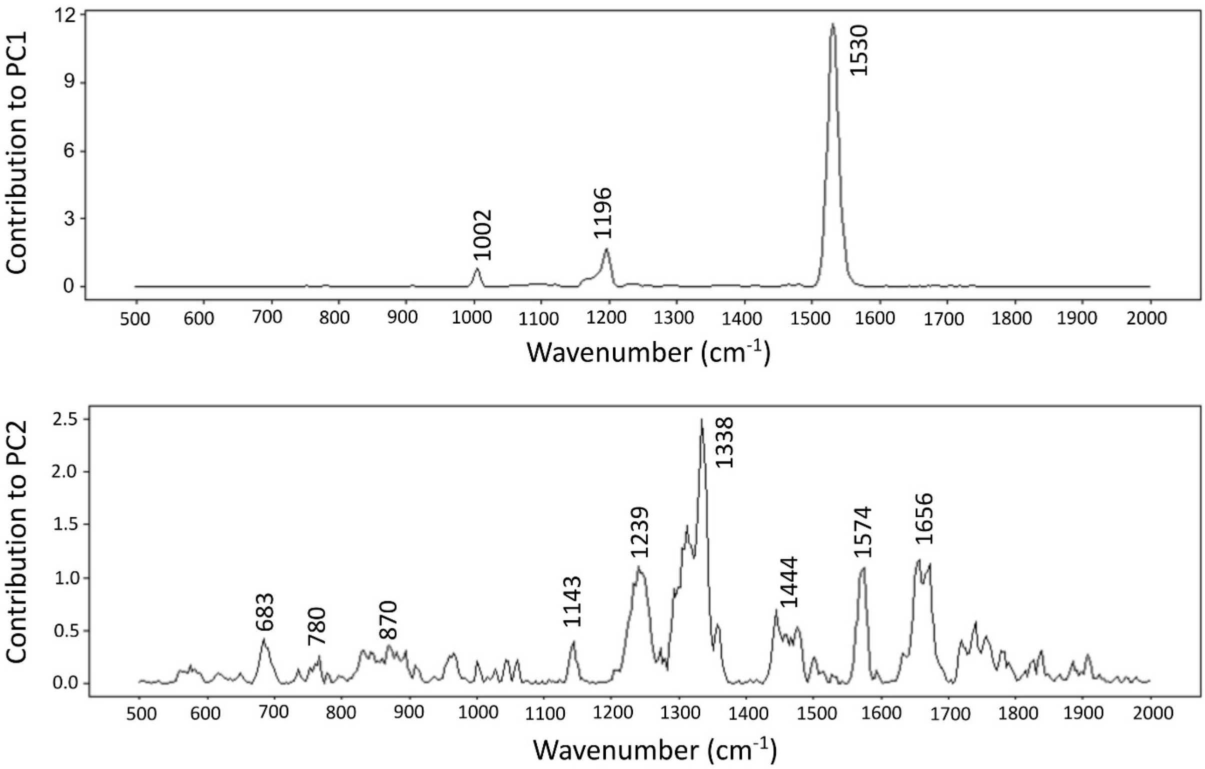

Fig S3 Loadings plot from principal component analysis showing the Raman spectral features that contribute to the PC1 and PC2 components.

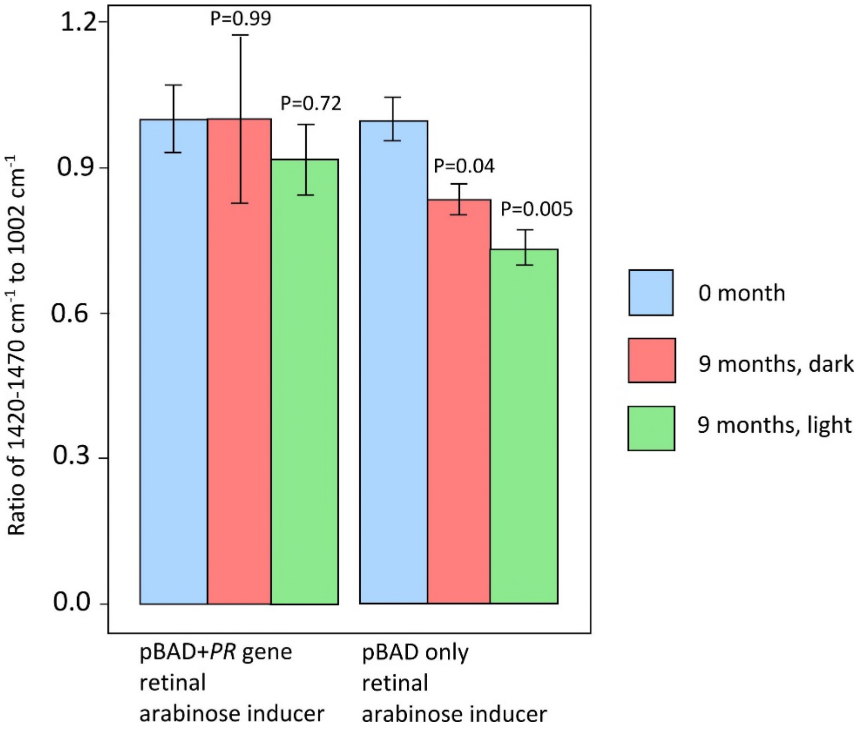

Fig S4 Comparison of PR vs non-PR cells on the basis of the ratio of 1420-1470 cm^{-1} signals, assigned to lipids, and normalised to the 1002 cm^{-1} signal for phenylalanine, taken here as a measure of protein content.

SUPPLEMENTAL FILE

TABLE S1 ^{14}N:^{15}N peptide isotopomer ion count ratios and copy numbers per cell.

TABLE S2 [^{14}N]PR peptide isotopomer series ion counts.

TABLE S3 [^{15}N]PR peptide isotopomer series ion counts.

TABLE S1 ^{14}N:^{15}N peptide isotopomer ion count ratios and copy numbers per cell.

Expressed PR was quantified by first converting the 200 pmol [^{15}N]PR-His$_6$ added to each assay to 1.20E+14 molecules and using this value multiplied by the mean of the ^{14}N:^{15}N peptide ion count ratios to calculate copy numbers per assay. This value was then divided by the number of cells per assay = 5.10E+8 ($\bar{x}_c$). Propagated error was calculated using $\sigma_c = \sqrt{5.10E+8} = 22583$ (Poisson statistics) according to the equation:

$\sigma_p = \bar{x}_p(\sqrt{(((\sigma_a / \bar{x}_a)^2) + ((\sigma_c / \bar{x}_c)^2)))}$

where $\bar{x}_a$, σ_a, $\bar{x}_p$ and σ_p are defined in the column headings.

Peptide	(D) pBAD+PR, +ara, +ret			Copies/assay		Copies/cell		(E) pBAD+PR, +ara, no ret			Copies/assay		Copies/cell	
	^{14}N:^{15}N			Mean ($\bar{x}a$)	SD (σa)	Mean ($\bar{x}p$)	Propagated	^{14}N:^{15}N			Mean ($\bar{x}a$)	SD (σa)	Mean ($\bar{x}p$)	Propagated e
	Rep 1	Rep 2	Rep 3	n = 12	n = 12	n = 12	n = 12	Rep 1	Rep 2	Rep 3	n = 12	n = 12	n = 12	n = 12
D.SPTVFRYI.D	0.70	0.75	0.77	9.53E+13	1.55E+13	186868	30333	0.75	0.66	0.67	7.54E+13	1.03E+13	147842	20113
D.SPTVFRYID.W	0.72	0.71	0.70					0.60	0.63	0.63				
D.GGSALNLNLIYNLA.D	1.10	0.64	0.75					0.72	0.70	0.59				
D.GGSALNLNLIYNLAD.F	0.91	0.90	0.88					0.50	0.45	0.64				

TABLE S2 [^{14}N]PR peptide isotopomer series ion counts.

Experiments are: (A) PR^{-} / L-arabinose^{-} / retinal^{-}, (B) PR^{-} / L-arabinose^{+} / retinal^{+}, (C) PR^{+} / L-arabinose^{-} / retinal^{-}, (D) PR^{+} / L-arabinose^{+} / retinal^{+} and (E), PR^{+} / L-arabinose^{+} / retinal^{-}. The sum of ^{14}N ion counts (from *E.* coli cells) for the three isotopomers for each peptide are shown.

Peptide	Isotopomer ions	(A) pBAD, no PR, no ara, no r			(B) pBAD, no PR, +ara, +re			(C) pBAD+PR, no ara, no ret			(D) pBAD+PR, +ara, +ret			(E) pBAD+PR, +ara, no ret		
		Ion count			Ion count			Ion count			Ion count			Ion count		
		Rep 1	Rep 2	Rep 3	Rep 1	Rep 2	Rep 3	Rep 1	Rep 2	Rep 3	Rep 1	Rep 2	Rep 3	Rep 1	Rep 2	Rep 3
	491.77															
D.SPTVFRYI.D	492.27	0	0	0	0	0	0	0	0	0	654415	468308	592857	940711	854054	797244
	492.77															
	549.29															
D.SPTVFRYID.W	549.79	0	0	0	0	0	0	0	0	0	2908496	4473555	4632100	4392462	3438893	4207158
	550.29															
	716.89															
D.GGSALNLNLIYNLA.D	717.39	0	0	0	0	0	0	0	0	0	720573	468544	478616	311972	420016	441366
	717.89															
	774.41															
D.GGSALNLNLIYNLAD.F	774.91	0	0	0	0	0	0	0	0	0	1642784	1725330	1584157	1154869	1318052	901407
	775.41															

TABLE S3 [^{15}N]PR peptide isotopomer series ion counts.

Experiments are: (A) pR$^-$ / L-arabinose$^-$ / retinal$^-$, (B) pR$^-$ / L-arabinose$^+$ / retinal$^+$, (C) pR$^+$ / L-arabinose$^-$ / retinal$^-$, (D) pR$^+$ / L-arabinose$^+$ / retinal$^+$ and (E), pR$^+$ / L-arabinose$^+$ / retinal$^-$. The sum of ^{14}N ion counts (from *E.* coli cells) for the three isotopomers for each peptide are shown.

Peptide	Isotopomer ions	(A) pBAD, no PR, no ara, no ret			(B) pBAD, no PR, +ara, +ret			(C) pBAD+PR, no ara, no ret			(D) pBAD+PR, +ara, +ret			(E) pBAD+PR, +ara, no ret		
		Ion count			Ion count			Ion count			Ion count			Ion count		
		Rep 1	Rep 2	Rep 3	Rep 1	Rep 2	Rep 3	Rep 1	Rep 2	Rep 3	Rep 1	Rep 2	Rep 3	Rep 1	Rep 2	Rep 3
	497.26															
D.SPTVFRYI.D	497.76	277532	386560	512510	985553	1142437	1587925	1416007	1416608	1052114	937467	622497	772344	1248884	1292673	1196758
	498.26															
	555.27															
D.SPTVFRYID.W	555.77	2325161	2325465	2660469	6567657	5158695	6242746	7063126	6215786	6298200	4067225	6274135	6608597	7268862	5472801	6729726
	556.27															
	725.37															
D.GGSALNLNLIYNLA.D	725.87	769655	785277	341898	1068941	1100366	647285	619710	704766	894696	655726	728832	637288	432699	602229	744289
	726.37															
	783.38															
D.GGSALNLNLIYNLAD.F	783.88	1347541	1250458	969413	2706240	3059318	1464054	3244126	1830968	2847235	1809820	1914654	1796889	2318417	2897045	1413961
	784.38															
